# Multiple micronutrient supplements versus iron‐folic acid supplements and maternal anemia outcomes: an iron dose analysis

**DOI:** 10.1111/nyas.14756

**Published:** 2022-02-25

**Authors:** Filomena Gomes, Rina Agustina, Robert E. Black, Parul Christian, Kathryn G. Dewey, Klaus Kraemer, Anuraj H. Shankar, Emily R. Smith, Andrew Thorne‐Lyman, Alison Tumilowicz, Megan W. Bourassa

**Affiliations:** ^1^ The New York Academy of Sciences New York New York; ^2^ NOVA Medical School Lisbon Portugal; ^3^ Department of Nutrition, Faculty of Medicine Universitas Indonesia ‐ Dr Cipto Mangunkusumo General Hospital Jakarta Indonesia; ^4^ Human Nutrition Research Centre, Indonesian Medical Education and Research Institute, Faculty of Medicine Universitas Indonesia Jakarta Indonesia; ^5^ Johns Hopkins Bloomberg School of Public Health Baltimore Maryland; ^6^ University of California, Davis Davis California; ^7^ Sight and Life Foundation Basel Switzerland; ^8^ University of Oxford Oxford UK; ^9^ Summit Institute for Development Mataram Indonesia; ^10^ The George Washington University Washington DC; ^11^ The Bill & Melinda Gates Foundation Seattle Washington

**Keywords:** micronutrient supplements, iron, pregnancy, anemia, iron deficiency anemia

## Abstract

Antenatal multiple micronutrient supplements (MMS) are more effective than iron and folic acid (IFA) supplements in reducing adverse pregnancy outcomes. Questions remain, however, about the ability of MMS to prevent anemia as effectively as IFA, especially at a lower dose of daily iron and in areas of high anemia prevalence. Analyzing data from 11 trials from a recent Cochrane review, we compared MMS to IFA, delivering either 30 or 60 mg of iron, in sustaining hemoglobin and preventing third trimester anemia and iron deficiency anemia (IDA), accounting for daily iron dose, total supplemental iron intake, and baseline prevalence of anemia. There were no differences between MMS and IFA in third trimester hemoglobin concentration or risks of anemia or IDA by iron dose or total supplemental iron consumed. MMS providing 30 mg of iron was comparable to IFA with 60 mg of iron: mean hemoglobin difference of −0.26 g/L (95% CI: −1.41 to 0.89), risk ratios of 0.99 (95% CI: 0.92–1.07) for anemia, and 1.31 (95% CI: 0.66–2.60) for IDA. Baseline prevalence of anemia did not explain heterogeneity in findings. Compared to IFA, MMS results in comparable hemoglobin concentration and protection against anemia during pregnancy, independently of iron dose.

## Graphical abstract

The goal of our analysis was to address the possible concerns related to use of micronutrient supplements (MMS) with 30 mg of iron versus iron and folic acid supplements (IFA) with 60 mg of iron, with regard to maternal anemia outcomes in low‐ and middle‐income countries. Compared to IFA, MMS results in comparable hemoglobin concentration and protection against anemia during pregnancy, independently of iron dose.

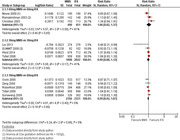

## Introduction

Multiple vitamin and mineral (micronutrient) deficiencies often coexist among women of reproductive age in low‐ and middle‐income countries (LMICs), especially during pregnancy when micronutrient requirements increase,^1^, ^2^ placing the health of the mother and child at risk.^3^ Multiple micronutrient supplements (MMS), that is, supplements providing several vitamins and minerals, can fill nutrient gaps for pregnant women and have been shown to be a safe and cost‐effective intervention to reduce adverse pregnancy and birth outcomes.^4^, ^5^, ^6^, ^7^ The most recent 2020 WHO guidelines on the use of MMS during pregnancy recommended this intervention “in the context of rigorous research”;^8^ this was an update to the “not recommended” decision from the 2016 WHO guidelines,^9^ wherein daily use of iron and folic acid (IFA) supplements was recommended.

Most prenatal MMS, including the widely used, researched, and well‐established United Nations International Multiple Micronutrient Antenatal Preparation (UNIMMAP) with 15 vitamins and minerals, contain 30 mg of elemental iron and 0.4 mg of folic acid.^10^ The WHO recommends antenatal IFA containing 30–60 mg of elemental iron to prevent maternal anemia,^9^ and states that 60 mg of iron is preferred^a^ in populations where anemia is a severe public health problem (i.e., in settings where at least 40% of pregnant women have a blood hemoglobin concentration <110 g/L).^9^ The conditional recommendation on the use of MMS from the 2020 WHO guidelines was based on a few concerns, including that “…more evidence is needed on the effects of switching to a 30 mg dose of iron from a higher dose of iron (e.g. 60 mg), particularly in settings where higher doses of iron are routinely used due to a high anaemia prevalence or other reasons.^8^ *”*

The impact of MMS and IFA on maternal anemia, among other outcomes, was analyzed in a Cochrane review (Keats *et al*.)^4^ that informed the 2020 WHO guideline development process. Nineteen studies conducted in LMICs that compared MMS (containing IFA) to iron supplements, with or without folic acid, were included. In the analysis that assessed the overall effect of MMS versus IFA on third trimester maternal anemia (hemoglobin <110 g/L), there was no difference between the groups (nine studies, RR 1.04, 95% CI 0.94–1.15). No subgroup analyses by different doses of iron, which varied from 20 to 60 mg, were conducted.

The 2020 WHO guideline^8^ separately assessed the effect of MMS and IFA on maternal anemia in the third trimester and conducted two subgroup analyses. The first subgroup compared MMS with any dose of iron versus IFA with 60 mg of iron and showed no difference in maternal anemia (seven trials, RR 1.04, 95% CI 0.90–1.21). The second subgroup compared MMS with any dose of iron versus IFA with 30 mg of iron, also showing no effect on anemia (RR 1.01, 95% CI 0.89–1.14), although only one study was eligible, demonstrating the limited evidence for inference. It should be noted that these analyses did not take into consideration the various levels of iron provided by MMS (20–60 mg). In addition, an analysis limited to the studies that provided MMS with the UNIMMAP formulation (MMS with 30 mg of iron versus IFA with 60 mg of iron) also showed no difference in maternal anemia between intervention and control (RR 0.90, 95% CI 0.77–1.05), although only two trials were included; there were no included studies comparing MMS with the UNIMMAP formulation versus IFA with 30 mg of iron.^8^

The MMS in Pregnancy Technical Advisory Group,^11^ hosted by New York Academy of Sciences, undertook a comprehensive analysis to examine the effect of MMS versus IFA on maternal anemia and iron status outcomes, including additional, previously unpublished data obtained from the clinical trial investigators. The goal of our analysis was to address the possible concerns related to the use of MMS with 30 mg of iron versus IFA with 60 mg of iron, with regard to maternal anemia outcomes in LMICs.^8^ We compared third trimester maternal anemia and iron status in response to MMS versus IFA supplementation stratified by different doses of iron in both formulations, by differences in total iron intake between IFA and MMS, and in relation to maternal baseline anemia levels.

## Methods

### Inclusion and exclusion criteria

Using the 19 studies included in the Cochrane review from Keats *et al*.,^4^ which compared the effect of prenatal MMS versus IFA on maternal, fetal, and infant health outcomes, we extracted data from the publications or follow‐up publications related to these studies for anemia and iron status. Our outcomes of interest were the prevalence of maternal anemia and iron deficiency anemia (IDA) and mean hemoglobin concentrations in the third trimester. The definitions of maternal anemia and IDA were the same as proposed in the parent trial. Most studies defined anemia (both at baseline and in the third trimester) as hemoglobin <110 g/L, and third trimester IDA as hemoglobin <110 g/L and serum ferritin <12 μg/L. Only one study (Ramakrishnan)^12^, ^13^ used slightly different cutoffs to define anemia (hemoglobin <110 g/L at baseline and <105 g/L in the third trimester) and IDA (hemoglobin <110 g/L at baseline and hemoglobin <105 g/L in the third trimester, in addition to serum ferritin <12 μg/L).

Exclusion criteria for this analysis were: (1) trials that did not assess third trimester iron‐related outcomes (e.g., assessed only postpartum); (2) trials with nondaily supplementation regimens; (3) trials conducted in high‐income countries (e.g., Brough);^14^ and (4) trials that provided MMS with 20 mg of iron (Ashorn^15^, ^16^ and Dewey^17^, ^18^), because this dose is not relevant for our comparison of interest, which is the difference between 60 mg of iron as used in many IFA supplementation programs and 30 mg dose of iron in MMS.

### Data extraction and management

When data were not available from the publications, authors were contacted and requested to analyze and provide the relevant data, via e‐mail.

Differences in total iron consumption from supplements by each group (IFA and MMS) over the course of each trial were estimated based on iron dose in each supplement, the duration and frequency of supplementation, as well as adherence information from each trial. Specifically, the duration of the supplementation period was calculated (in days) as the mean gestational age at third trimester assessment (in weeks) minus the mean gestational age at enrollment (in weeks) and multiplied by the number of days of supplementation per week. This was then multiplied by the amount of iron provided in each tablet (in mg) and the adherence (percent adherence/100).

As an example, for the Christian 2003 study, the supplementation period of interest was defined from inclusion (mean of 11.6 weeks of gestation in the MMS arm and 11.3 weeks in the IFA arm) until 32 weeks of gestation, when the third trimester hemoglobin assessment was performed. With a daily supplementation regimen (7 days per week), the supplementation period of interest (i.e., maximum number of days that the supplements could have been consumed) was 142.8 days in the MMS arm and 144.9 days in the IFA arm. The MMS group received 60 mg of iron per day and had a mean adherence rate of 76.2% (percentage of all eligible doses consumed), accounting for a total amount of iron consumed of 6529 mg (i.e., 60 mg of iron × 142.8 days × 0.762). The IFA group, which also received 60 mg of iron per day, had a mean adherence rate of 74.9%, accounting for a total amount of iron consumed of 6512 milligrams.

The difference in the estimated total iron consumption between the IFA and MMS arms was calculated and then categorized into small (if less than 1200 mg) or large (if greater than 1200 mg), for the purpose of the subgroup analysis by differences in total iron intake between IFA and MMS. The cutoff of 1200 mg was chosen after examining the distribution of differences in total iron intake.

We assessed the effect of MMS versus IFA on third trimester maternal anemia and iron status in: (1) subgroup analyses by daily total supplemental iron intake (IFA with 60 mg of iron versus MMS with 60 mg of iron; IFA with 30 mg of iron versus MMS with 30 mg of iron; IFA with 60 mg of iron versus MMS with 30 mg of iron); and (2) subgroup analyses by differences in total iron intake between IFA and MMS (small difference IFA‐MMS (<1200 mg) and large difference IFA‐MMS (≥1200 mg)). In addition, we performed meta‐regressions to assess potential effect modification by baseline anemia prevalence in the study population. While we included studies providing different doses of iron in the MMS and IFA groups, the most important comparison and, therefore the focus of our analysis, is the comparison of MMS providing 30 mg of iron versus IFA providing 60 mg of iron. This is the comparison that directly addresses the concern expressed in the 2020 WHO Guideline.

### Cluster‐randomized trials

In our analyses, we included five cluster‐randomized trials (Christian;^19^ SUMMIT;^20^ Sunawang;^21^ West;^22^ and Zeng).^23^ Meta‐analysis pooling the results of cluster randomized trials with individually randomized trials requires an adjustment for the design effect of the cluster design. Because the five trials did not present the design effects or intracluster coefficients needed for the specific outcomes presented in this analysis, we applied the design effects presented for other outcomes presented in Table S1 (online only) to the number of events and sample sizes for dichotomous outcomes and to the sample sizes for continuous outcomes, in order to reduce cluster‐randomized trial data to their effective sample size. Effect estimates and 95% confidence intervals, adjusted for design effect, were then calculated and analyzed with individually randomized trials using the generic inverse variance (DerSimonian and Laird)^24^ method for the analyses that included cluster‐randomized trials.^25^

### Trials with multiple intervention groups

In the following trials with multiple intervention groups, we selected the most relevant comparison groups (intervention and control) for this analysis and excluded the other group(s), as proposed by the *Cochrane Handbook for Systematic Reviews of Interventions*:^26^

- For Christian,^19^ we included data from group 4 (MMS) versus group 2 (iron, folic acid, and vitamin A), excluding groups 1 (folic acid with vitamin A), 3 (iron, folic acid, vitamin A, and zinc), and 5 (vitamin A only).
- For Liu^27^ and Zeng,^23^ we included data from group 3 (MMS) versus group 2 (IFA), and excluded group 1 (folic acid only).
- For Moore,^28,29^ we included data from group 2 (MMS) and group 1 (IFA), excluding group 3 (food‐based supplement providing protein‐energy plus IFA) and group 4 (food based supplement providing protein‐energy plus MMS with IFA).

For Tofail^30^, ^31^ (with six arms), we used a different approach that is also recommended by the *Cochrane Handbook for Systematic Reviews of Interventions*.^26^ This trial had three “early invitation to food” arms and three “usual invitation to food” arms that included IFA and MMS. First, we combined the “early invitation” and “usual invitation to food” groups to create a single pairwise comparison, to compare MMS with 30 mg of iron versus IFA with 30 mg of iron, and MMS with 30 mg of iron versus IFA with 60 mg of iron in the subgroup analyses. For the estimation of the overall effect of MMS versus IFA (i.e., in the meta‐regressions), we split the shared group (MMS with 30 mg of iron) into two groups, whereby the number of events and sample sizes of that arm were halved for the dichotomous outcomes and only the total number of participants was divided for continuous variables.

### Data analysis and meta regression

Review Manager (version 5.4) was used to calculate the pooled effect estimates of MMS versus IFA with risk ratios (RR), mean differences, and risk differences for the outcomes of interest. In this meta‐analysis, we pooled together data from individually randomized and cluster‐randomized trials, using the generic inverse variance (DerSimonian and Laird)^24^ method after adjustment of estimates from cluster‐randomized trials, as noted above.

Meta‐regressions were used to explore whether heterogeneity in findings related to the effect of MMS versus IFA on third trimester maternal anemia and iron status was explained by baseline maternal anemia. The metareg package in Stata 16.1 was used with a Hartung–Knapp variance estimator and associated *t*‐test to determine *P* values. A *P* value < 0.05 was considered statistically significant for all analyses.

## Results

The list of trials considered for inclusion and the reasons for exclusion from these iron dose analyses is presented in Table S2 (online only). We screened 19 studies, after which a total of 11 trials were included and contributed to the analyses of the three outcomes of interest. One trial^32^ was excluded because of the intermittent (twice weekly) supplementation scheme, four trials^33^, ^34^, ^35^, ^36^ were excluded because hemoglobin was not assessed in the third trimester, two trials^15^, ^16^, ^17^, ^18^ were excluded because MMS provided 20 mg of iron, and one trial^37^ was excluded because of lack of relevant information, for example, the amount of iron provided in both groups was not available.

From the 11 trials included in the present analyses, some assessed maternal anemia and iron status in a proportion of the whole study population, that is, Liu,^27^, ^38^ Ramakrishnan,^12^ SUMMIT,^20^ and Zeng^23^ assessed the third trimester hemoglobin levels in approximately 5%, 52%, 0.7%, and 10% of the study sample, respectively.

The included studies varied with regard to iron dose in each supplement (from 30 to 60 mg), frequency of supplementation (from 6 to 7 days a week), duration of supplementation (from enrollment, which varied from 9 to 24 weeks of gestation, until delivery), and adherence levels (which were measured in different ways, e.g., proportion of consumed tablets and number of supplements taken). The estimated total iron consumption in each study arm, from randomization until the third trimester assessment of maternal anemia and iron status, took into account all variables and is presented in Table 1.

**Table 1:** Overview of total supplemental iron intake

Study author, year	Iron dose in MMS arm (mg)	Iron dose in IFA arm (mg)	Total amount of iron consumed (mg)–MMS arm	Total amount of iron consumed (mg)–IFA arm	Difference between IFA and MMS (mg)
Christian,^19^ 2003	60	60	6529	6512	−17
Liu,^27^ 2013	30	30	3536	3586	50
Moore,^28^ 2009	60	60	6374	6511	138
Osrin,^40^ 2005	30	60	3157	6215	3058
Ramakrishnan,^12^ 2003	60	60	7832	7729	−103
Roberfroid,^39^ 2008	30	60	1328	2545	1218
SUMMIT,^20^ 2008	30	30	3226	3034	−191
Sunawang,^21^ 2009	30	60	2513	5248	2735
Tofail,^30^ 2008 (IFA 30 mg)	30	30	2250	2370	120
Tofail,^30^ 2008 (IFA 60 mg)	30	60	2250	4680	2430
West,^22^ 2014	27^*a*^	27^*a*^	3780	3780	0
Zeng,^23^ 2008	30	60	3131	6330	3199
Minimum			1328	2370	
Maximum			7832	7729	

^*a*^ Both arms of the West 2014 trial received a supplement that contained 27 mg of iron. However, the analysis of chemical composition of MMS and IFA tablets showed that the intended iron in MMS (27 mg) varied from 105 to 112%, which is equivalent to 28.4–30.2 mg of iron, and the intended iron in IFA (27 mg) varied from 97 to 111%, which is equivalent to 26.2–29.8 mg of iron. Thus, for the purpose of our subgroup analyses by iron dose, we considered this study as providing 30 mg of iron in each study arm.

The anemia prevalence and mean hemoglobin levels for each study and study arms are presented in Table 2. The proportion of anemia at baseline varied from 5%^27^, ^38^ to 46.8%.^20^ Two studies, conducted in Indonesia^20^ and Burkina Faso,^39^ had a mean baseline anemia prevalence over 40%.

**Table 2:** Baseline characteristics: anemia prevalence and hemoglobin levels

Study author, year	Country	Proportion of anemic women at baseline (%)–average of proportions in each study arm	Hemoglobin at baseline (g/L)–average of mean values in each study arm
Christian,^19^ 2003	Nepal	33.3	115.0
Liu,^27^ 2013	China	5.0	122.0
Moore,^28^ 2009	The Gambia	36.4	114.3
Osrin,^40^ 2005	Nepal	38.0	115.0
Ramakrishnan,^12^ 2003	Mexico	14.2	125.5
Roberfroid,^39^ 2008	Burkina Faso	46.5	110.0
SUMMIT,^20^ 2008	Indonesia	46.8	109.7
Sunawang,^21^ 2009	Indonesia	37.5	113.0
Tofail,^30^ 2008 (IFA 30 mg)	Bangladesh	28.5	116.9
Tofail,^30^ 2008 (IFA 60 mg)	Bangladesh	28.5	116.6
West,^22^ 2014	Bangladesh	21.7	117.6
Zeng,^23^ 2008	China	N.A.	N.A.

note: The gestational time of baseline hemoglobin/anemia assessment (gestational age at enrollment) varied from 9 weeks in the trial West 2014 to 24 weeks in the trial Roberfroid.^39^

N.A., not available.

The summaries of results are presented in Tables 3, 4, 5 and include the effect estimates obtained from the subgroup analyses by iron dose and differences in total iron intake between IFA and MMS. The forest plots for the subgroup analysis by different iron doses, for maternal anemia, maternal hemoglobin, and maternal IDA, are presented in Figures S1–S3 (all online only), respectively.

**Table 3:** Summary of results: subgroup analyses for maternal anemia

	Random effects model			Random effects model			
Effect of MMS versus IFA on maternal anemia (third trimester), Hb<110 g/L for most studies	*n* comparisons	Risk ratio (95% CI)	*P* value (subgroup differences)	*n* comparisons	Risk difference (95% CI)	*P* value (subgroup differences)	Included studies
Subgroup analysis by iron dose							
MMS 60 mg iron versus IFA 60 mg iron	3	1.06 (0.82−1.37)	0.89	3	0.06 (−0.20 to 0.31)	0.89	12, 19, 28
MMS 30 mg iron versus IFA 30 mg iron	4	0.99 (0.88−1.12)		4	−0.01 (−0.13 to 0.12)		20, 22, 27, 30
MMS 30 mg iron versus IFA 60 mg iron	5	0.99 (0.92−1.07)		5	−0.01 (−0.08 to 0.07)		21, 23, 30, 39, 40
Subgroup analysis by differences in total iron intake between IFA and MMS							
Small difference IFA‐MMS (<1200 mg)	7	1.01 (0.90−1.12)	0.84	7	0.01 (−0.10 to 0.12)	0.84	12, 19, 22, 27, 28, 30
Large difference IFA‐MMS (≥1200 mg)	5	0.99 (0.92−1.07)		5	−0.01 (−0.08 to 0.07)		21, 23, 30, 39, 40

Christian;^19^ Liu;^27^ Moore;^28^ Osrin;^40^ Ramakrishnan;^12^ Roberfroid;^39^ SUMMIT;^20^ Sunawang;^21^ Tofail;^30^ West;^22^ and Zeng.^23^

**Table 4:** Summary of results: subgroup analyses for maternal hemoglobin

	Random effects model			
Effect of MMS versus IFA on **hemoglobin (third trimester), g/L**	*n* comparisons	Mean difference (95% CI)	*P* value (subgroup differences)	Included studies
Subgroup analysis by iron dose				
MMS 60 mg iron versus IFA 60 mg iron	3	−0.68 (−3.56, 2.20)	0.78	12, 19, 28
MMS 30 mg iron versus IFA 30 mg iron	4	0.14 (−0.71, 0.99)		20, 22, 27, 30
MMS 30 mg iron versus IFA 60 mg iron	4	−0.26 (−1.41, 0.89)		21, 23, 30, 40
Subgroup analysis by differences in total iron intake between IFA and MMS				
Small difference IFA‐MMS (<1200 mg)	7	0.17 (−0.85, 1.20)	0.58	12, 19, 20, 22, 27, 28, 30
Large difference IFA‐MMS (≥1200 mg)	4	−0.26 (−1.41, 0.89)		21, 23, 30, 40

Christian;^19^ Liu;^27^ Moore;^28^ Osrin;^40^ Ramakrishnan;^12^ SUMMIT;^20^ Sunawang;^21^ Tofail;^30^ West;^22^ and Zeng.^23^

**Table 5:** Summary of results: subgroup analyses for maternal iron deficiency anemia

	Random effects model			Random effects model			
Effect of MMS versus IFA on iron deficiency anemia (third trimester), Hb<110 g/L and serum ferritin<12 μg/dL for most studies	*n* comparisons	Risk ratio (95% CI)	*P* value (subgroup differences)	*n* comparisons	Risk difference (95% CI)	*P* value (subgroup differences)	Included studies
Subgroup analysis by iron dose							
MMS 60 mg iron versus IFA 60 mg iron	3	1.18 (0.94−1.48)	0.76	3	0.17 (−0.06 to 0.39)	0.76	12, 19, 28
MMS 30 mg iron versus IFA 30 mg iron	2	0.91 (0.43−1.93)		2	−0.10 (−0.85 to 0.66)		27, 30
MMS 30 mg iron versus IFA 60 mg iron	2	1.31 (0.66−2.60)		2	0.27 (−0.41 to 0.96)		21, 30
Subgroup analysis by differences in total iron intake between IFA and MMS							
Small difference IFA‐MMS (<1200 mg)	5	1.15 (0.93−1.41)	0.71	5	0.14 (−0.07 to 0.35)	0.71	12, 19, 27, 28, 30
Large difference IFA‐MMS (≥1200 mg)	2	1.31 (0.66−2.60)		2	0.27 (−0.41 to 0.96)		21, 30

Christian;^19^ Liu;^27^ Moore;^28^ Ramakrishnan;^12^ Sunawang;^21^ and Tofail.^30^

For the outcome of third trimester maternal anemia, we included more studies (11 trials with 9638 participants) than the analyses previously conducted by Keats *et al*.^4^ (nine trials with 5912 participants) and WHO^8^ (eight trials with an unknown number of participants). The subgroup analyses by iron dose or differences in total iron intake showed no differences between MMS and IFA in any of the subgroups, for the three outcomes (Tables 3, 4, 5). For our main comparison of interest, MMS providing 30 mg of iron did not result in an increased risk of anemia (five trials, 4677 participants; RR 0.99, 95% CI 0.92–1.07), nor lower levels of hemoglobin (four trials, 3882 participants; mean difference −0.26, 95% CI −1.41 to 0.89) or increased risk of IDA (two trials, 590 participants; RR 1.31, 95% CI 0.66–2.60), when compared to IFA providing 60 mg of iron.

Table S3 (online only) shows the results of the meta‐regressions conducted to assess whether the baseline anemia prevalence is associated with the overall effect of MMS versus IFA on third trimester maternal anemia, hemoglobin levels, or IDA. Results suggest that this factor does not explain heterogeneity in findings of the trials for any of these outcomes.

## Discussion

These analyses reveal that, in LMIC where 5% to nearly 50% of women are anemic early in pregnancy, antenatal MMS providing 30 or 60 mg of iron results in comparable maternal hemoglobin concentrations and protection against anemia, including that attributed to iron deficiency, as IFA providing the same levels of iron. Especially noteworthy is the observation that MMS delivering 30 mg of iron is comparable to IFA with 60 mg of iron with regard to these outcomes.

For our main comparison of interest (MMS with 30 mg of iron versus IFA with 60 mg of iron), five trials with 4677 participants contributed to the estimated effect size (RR) for maternal anemia of 0.99, 95% CI 0.92–1.07, suggesting that providing MMS with 30 mg of iron during pregnancy is comparable to IFA with 60 mg of iron in terms of preventing third trimester anemia. Furthermore, these five trials had relatively high levels of baseline anemia, that is, 29% for Tofail *et al*.,^30^, ^31^ 38% for Osrin *et al*.^40^ and Sunawang,^21^ 47% for Roberfroid *et al*.,^39^ and unknown levels for Zeng *et al*.,^23^ suggesting that the transition from IFA with 60 mg of iron to MMS with 30 mg of iron in areas with a high prevalence of anemia can occur without elevating the risk of maternal anemia in the third trimester of gestation. However, the number of studies that assessed IDA was very limited (two trials with only 590 participants), making it difficult to draw firm conclusions about that outcome.

Meta‐regressions suggested that baseline anemia prevalence did not modify the results comparing MMS versus IFA for any of the outcomes, although the number of studies contributing to those analyses was relatively small, particularly for the outcome of IDA, which included only seven trials.

Our findings are consistent with the results of a randomized controlled trial designed to determine the lowest dose of iron to prevent iron deficiency (serum ferritin <13 μg/L) and IDA (serum ferritin <13 μg/L and hemoglobin <5th percentile) in pregnancy in a high‐income country.^41^ In that study, 427 healthy (nonanemic) Danish pregnant women were allocated into four groups receiving ferrous iron in doses of 20, 40, 60, and 80 mg from 18 weeks of gestation.^41^ While 20 mg ferrous iron was an inadequate dose to use as a prophylaxis against iron deficiency, a dose of 40 mg ferrous iron per day (taken from 18 weeks of pregnancy) was adequate as it prevented anemia in 90% of the women and IDA in over 95% of the pregnant and postpartum women. Although this study was conducted in a high‐income country, which limits the comparability with the LMIC trials included in our analyses, it is estimated that approximately 40% of Danish women of reproductive age have low body iron reserves (i.e., serum ferritin lower than 30 μg/L) and, in this study, 50.7% of the women had ferritin levels lower than 30 μg/L at 18 weeks of gestation.^41^ Likewise, similar results for anemia reduction have been observed in a trial conducted in Australia, wherein anemic pregnant women (with hemoglobin levels <110 g/L in mid‐pregnancy) received doses of 20, 40, or 80 mg of iron for 8 weeks.^42^ At the end of the intervention, the incidence of anemia did not differ between groups (38%, 26%, and 24%, respectively). When compared to those receiving 80 mg of iron, the risk of developing IDA was higher in women receiving 20 mg of iron but not in women receiving 40 mg of iron.

The observation that 30 mg of iron in MMS is comparable to 60 mg of iron in IFA in preventing third trimester anemia is, therefore, plausibly explained, in part, by three physiological mechanisms. First, the presence of other micronutrients, such as vitamin A, vitamin C, and riboflavin, can improve the absorption and/or utilization of iron compared with the iron alone.^3^ Second, other micronutrients in MMS, such as vitamins B12 and vitamin A, may ameliorate key deficiencies known to cause anemia in pregnant women.^43^, ^44^ In fact, combinations of these nutrients can synergize with iron to prevent maternal anemia, as demonstrated in a trial of vitamin A and iron supplementation conducted in Indonesia.^45^ In that trial of 251 anemic pregnant women, the proportion of women who became nonanemic was only 16% in the placebo group, compared to 35% in the vitamin‐A–supplemented group, 68% in the group that received iron supplements, and 97% in the group supplemented with both iron and vitamin A. Third, a recent review of stable isotope absorption studies of oral iron supplementation for women with iron deficiency, or IDA, concluded that daily oral doses ≤40 mg were preferred. This was because iron doses ≥ 60 mg can trigger a transient increase in circulating hepcidin that can inhibit iron absorption, a phenomenon not seen with iron doses ≤40 mg.^46^

It is important to note that MMS and IFA are used for the *prevention* of anemia and other adverse outcomes in pregnancy, for which 30 mg of iron per day is most likely an adequate dose.^4^, ^8^ When IDA is already present at entry to antenatal care or develops during pregnancy, it would be reasonable to include additional iron supplementation while continuing use of MMS. However, clinical and laboratory assessment should be conducted to determine if the anemia is primarily due to iron deficiency or to other causes, such as other micronutrient deficiencies (e.g., vitamins A, folic acid, and B12 deficiency), genetic hemoglobin disorders, inflammation, or infectious diseases (e.g., tuberculosis, HIV, and parasitic infections).^47^ If iron deficiency is not the main cause of anemia, more iron would not reduce anemia and would be an unnecessary intervention with the potential for increasing the risk of harmful effects.^48^, ^49^, ^50^, ^51^, ^52^, ^53^, ^54^, ^55^, ^56^ A study conducted in rural Bangladeshi women (*n* = 207) showed that the high prevalence of thalassemia (27%) contributed to the risk of anemia, unlike iron deficiency, which was absent most likely due to high iron intake from groundwater.^57^ Thus, for each setting, it is crucial to understand the environmental sources of iron, as well as the role of hemoglobinopathies and other factors contributing to anemia at the population level.

The present analyses were focused on hematologic outcomes and, as such, we did not examine the effect of different doses of iron on other maternal and birth outcomes. However, Keats *et al*.^4^ performed similar iron dose analyses for the outcomes preterm births, small‐for‐gestational age, and perinatal mortality and did not find differences between MMS and IFA, except for the following subgroups, which favored MMS: MMS with 30 mg of iron versus IFA with 60 mg of iron resulted in a reduction of risk of small‐for‐gestational age (RR 0.89, 95% CI 0.81–0.97), MMS with 30 mg of iron versus IFA with 30 mg of iron resulted in a reduction of risk of small‐for‐gestational age (RR 0.98, 95% CI 0.96–1), and perinatal mortality (RR 0.92, 95% CI 0.86–0.98).

In summary, when compared to IFA, MMS is known to have additional benefits in the risk reduction of stillbirth, infant mortality at 6 months, low birthweight, preterm birth, and being born small‐for‐gestational age, with greater risk reductions among anemic pregnant women (as described by Smith and colleagues),^5^ and our data suggest that transition from IFA with 30 or 60 mg of iron to MMS with 30 mg of iron would not increase the risk of maternal anemia.

### Limitations

There are a few limitations of these analyses. First, despite our success in obtaining data on anemia and iron status from all the studies that assessed these same outcomes during the third trimester, some trials (Liu,^27^, ^38^ Ramakrishnan,^12^ SUMMIT,^20^ and Zeng)^23^ only assessed iron‐related outcomes in a proportion of the whole study population, which may introduce a potential selection bias if subsamples were not selected randomly. The subsample of the study population that contributed to the maternal anemia and iron status analysis was randomly selected for three of the trials (Liu,^27^, ^38^ SUMMIT,^20^ and Zeng),^23^ while it seemed to be a convenience sample (based on available data from blood collected at three time points) for one of them (Ramakrishnan).^12^ However, in the latter case, the authors compared this subsample (*n* = 453) to those with incomplete blood data and/or those who were lost to follow‐up (*n* = 420) and did not find any differences in baseline characteristics, with the exception that women in the subsample were less likely to be primiparous. Second, the cutoffs used to define third trimester anemia and IDA were the same for most of the studies, but not all, which limits the comparability between studies. It should also be noted that work is underway to determine whether the current cutoffs to define anemia during pregnancy are too high,^58^ as suggested by a recent study that generated international, gestational age‐specific smoothed centiles for optimal maternal hemoglobin concentration.^59^ Third, our results are derived from LMIC, and their applicability to high‐income countries or populations not at risk of micronutrient deficiencies is not known.

## Conclusion

The present work provided a comprehensive overview of the existing evidence regarding the effect of MMS versus IFA containing different doses of iron on maternal anemia and iron‐related outcomes in LMICs. We gathered published and unpublished data (provided directly by the study authors) from the 11 studies identified by systematic literature searches conducted by a recent Cochrane review^4^ that assessed third trimester hematological outcomes, and obtained the effect estimates for at least one of our outcomes of interest from all of these studies.

The overall effect of MMS versus IFA on third trimester maternal anemia and iron‐related outcomes does not seem to be modified by baseline levels of anemia. We also found no differences for any of the outcomes when the analysis was limited to the studies that provided MMS with 30 mg of iron versus IFA with 60 mg of iron. However, the number of studies contributing data on IDA for this comparison was limited, and additional research is needed to draw more firm conclusions for this outcome. Clinical trials assessing the effect of different doses of iron in MMS, or in addition to MMS, on maternal IDA in populations with high baseline levels of anemia, would help to fill this data gap. Because MMS with 30 mg of iron influenced hemoglobin with clinically comparable results to IFA with 60 mg iron, and because MMS significantly improves fetal growth and survival, especially in anemic women,^3^, ^4^, ^5^, ^6^, ^7^ we suggest that policymakers in LMIC proceed with the transition from IFA to MMS. However, this transition should be informed by implementation research designed to optimize MMS introduction (and compliance) and continuing clinical research that will inform future WHO guidelines as they are updated.

## Competing interests

The authors declare no competing interests.

### Peer review

The peer review history for this article is available at: https://publons.com/publon/10.1111/nyas.14756

## Supporting information


**Figure S1**. Effect of MMS versus IFA on maternal anemia: subgroup analysis by iron dose.

**Figure S2**. Effect of MMS versus IFA on maternal hemoglobin: subgroup analysis by iron dose.

**Figure S3**. Effect of MMS versus IFA on maternal iron deficiency anemia: subgroup analysis by iron dose.

**Table S1**. Estimated design effects of the five cluster‐randomized controlled trials.

**Table S2**. List of included and excluded trials, study design characteristics, and outcomes.

**Table S3**. Summary of results: meta‐regressions of the effect of MMS versus IFA according to baseline anemia prevalence.

## Footnotes

a The recommendation on MMS from the 2020 WHO guideline includes the following remark: “(…) WHO *recommends* antenatal iron and folic acid supplements containing 60 mg of elemental iron in populations where anaemia is a severe public health problem.” As a remark—which is based on expert opinion, rather than evidence based—this statement should be interpreted with caution. The document supporting this statement (http://apps.who.int/iris/bitstream/handle/10665/259425/9789241513067‐eng.pdf) is a WHO reference document, which states that daily dose of 60 mg of iron is *preferred* in areas where at least 40% of pregnant women are anemic.
