# Supplementary figures and images for: Multiple micronutrient supplements versus iron‐folic acid supplements and maternal anemia outcomes: an iron dose analysis
Source: Ann N Y Acad Sci. 2022 Feb 25;1512(1):114–25. doi: 10.1111/nyas.14756 (PMC9306935; doi:10.1111/nyas.14756)

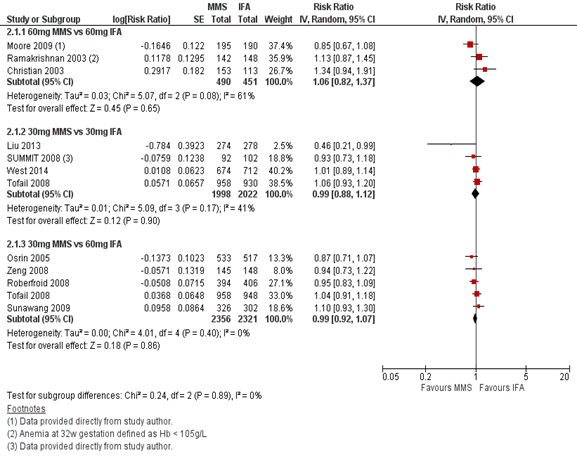

Supplement: Supplementary file 1 — Figure S1. Effect of MMS versus IFA on maternal anemia: subgroup analysis by iron dose. [file NYAS-1512-114-s001.png]

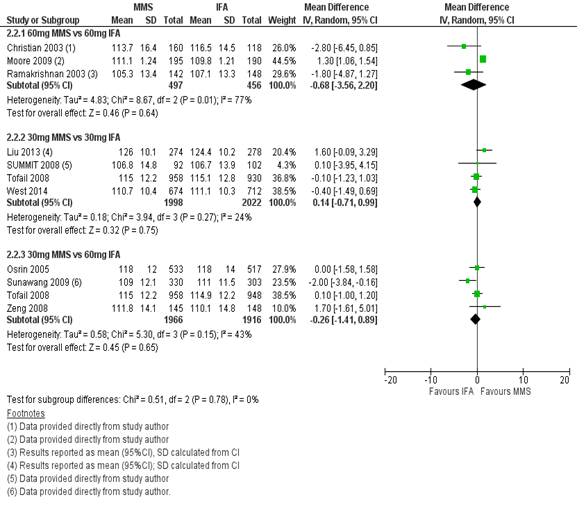

Supplement: Supplementary file 2 — Figure S2. Effect of MMS versus IFA on maternal hemoglobin: subgroup analysis by iron dose. [file NYAS-1512-114-s006.png]

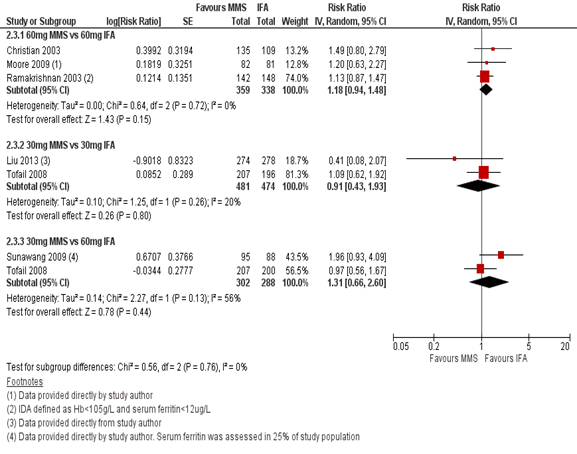

Supplement: Supplementary file 3 — Figure S3. Effect of MMS versus IFA on maternal iron deficiency anemia: subgroup analysis by iron dose. [file NYAS-1512-114-s005.png]
