# Supplementary material for: Multiple micronutrient supplements versus iron‐folic acid supplements and maternal anemia outcomes: an iron dose analysis
Source: Ann N Y Acad Sci. 2022 Feb 25;1512(1):114–25. doi: 10.1111/nyas.14756 (PMC9306935; doi:10.1111/nyas.14756)
Supplement: Supplementary file 4 — Table S1. Estimated design effects of the five cluster‐randomized controlled trials. [file NYAS-1512-114-s003.docx]

### Appendix Table 1 – Estimated design effects of the five cluster-randomized controlled trials

| **Trial** | **Estimated design effect** |
| --- | --- |
| Christian 2003^19^ | 1.2 |
| SUMMIT 2008^20^ | 1.2 |
| Sunawang 2009^21^ | 1.2 |
| West 2014^22^ | 1.15 |
| Zeng 2008^23^ | 1.3 |
