# Supplementary material for: Multiple micronutrient supplements versus iron‐folic acid supplements and maternal anemia outcomes: an iron dose analysis
Source: Ann N Y Acad Sci. 2022 Feb 25;1512(1):114–25. doi: 10.1111/nyas.14756 (PMC9306935; doi:10.1111/nyas.14756)
Supplement: Supplementary file 5 — Table S2. List of included and excluded trials, study design characteristics, and outcomes. [file NYAS-1512-114-s004.docx]

### Appendix Table 2 – List of included and excluded trials, study design characteristics and outcomes

| **Study author, year*** | **Included/excluded, reason for exclusion** | **Cluster RCT?** | **Multiple intervention groups?** | **Available outcomes** | **Data provided directly by study authors** |
| --- | --- | --- | --- | --- | --- |
| Ashorn, 2015^15,16^ | Excluded because MMS provided 20 mg of iron | - | - | - | - |
| Bhutta, 2009^33^ | Excluded because iron outcomes were assessed at first postnatal visit, not in the third trimester | - | - | - | - |
| Biggs, 2010^32^ | Excluded because of the twice weekly supplementation | - | - | - | - |
| Christian, 2003^19^ | Included | Yes | Yes | Anemia, hemoglobin, iron deficiency anemia | Hemoglobin (mean, SD, number of participants) |
| Dewey, 2009^17,18^ | Excluded because MMS provided 20 mg of iron | - | - | - | - |
| Fawzi, 2007^34^ | Excluded because iron outcomes were assessed 6 weeks post-delivery, not in the third trimester | - | - | - | - |
| Friis, 2004^37^ | Excluded because too little information is available on iron dose and iron outcomes were not assessed in the third trimester | - | - | - | - |
| Kaestel, 2005^35^ | Excluded because iron outcomes were assessed 2 months post-delivery, not in the third trimester | - | - | - | - |
| Liu, 2013^27,38^ | Included | No | Yes | Anemia, hemoglobin, iron deficiency anemia | Iron deficiency anemia (number of events an participants) |
| Moore, 2009^28,29^ | Included | No | Yes | Anemia, hemoglobin, iron deficiency anemia | All outcomes (mean, SD, number of events and participants), adherence rates and definition |
| Osrin, 2005^40^ | Included | No | No | Anemia, hemoglobin |  |
| Ramakrishnan, 2003^12,13^ | Included | No | No | Anemia, hemoglobin, iron deficiency anemia |  |
| Roberfroid, 2008^39^ | Included | No | No | Anemia | Anemia (number of events an participants) provided by Emily Keats^4^ |
| SUMMIT, 2008^20^ | Included | Yes | No | Anemia, hemoglobin | Both outcomes (mean, SD, number of events and participants, for first and second trimester enrollees who had a third trimester blood assessment) |
| Sunawang, 2009^21^ | Included | Yes | No | Anemia, hemoglobin, iron deficiency anemia | All outcomes and baseline anemia levels (mean, SD, number of events and participants) |
| Tofail, 2008^30,31^ | Included | No | Yes | Anemia, hemoglobin, iron deficiency anemia | All outcomes and baseline anemia levels (mean, SD, number of events and participants) |
| West, 2014^22^ | Included | Yes | No | Anemia, hemoglobin |  |
| Zagre, 2007^36^ | Excluded because hemoglobin was not assessed at baseline or follow-up | - | - | - | - |
| Zeng, 2008^23^ | Included | Yes | No | Anemia, hemoglobin |  |

** as identified in the Cochrane review (Keats et al, 2019)^4^, although other follow-up publications were used for the present iron dose analyses*

*RCT = randomized controlled trial, SD = Standard deviation*
