# Supplementary material for: Multiple micronutrient supplements versus iron‐folic acid supplements and maternal anemia outcomes: an iron dose analysis
Source: Ann N Y Acad Sci. 2022 Feb 25;1512(1):114–25. doi: 10.1111/nyas.14756 (PMC9306935; doi:10.1111/nyas.14756)
Supplement: Supplementary file 6 — Table S3. Summary of results: meta‐regressions of the effect of MMS versus IFA according to baseline anemia prevalence. [file NYAS-1512-114-s002.docx]

### Appendix Table 3 - Summary of results: meta-regressions of the effect of MMS vs IFA according to baseline anemia prevalence

| **Explanatory variable** | **Outcome variable** | **Number of studies** | **Regression coefficient** | **P value** |
| --- | --- | --- | --- | --- |
| Baseline maternal anemia prevalence | Third trimester maternal anemia (risk ratio) | 11 | 1.00 | 0.47 |
|  | Third trimester maternal hemoglobin levels, g/L (mean difference) | 10 | -0.02 | 0.61 |
|  | Third trimester iron deficiency anemia (risk ratio) | 7 | 1.01 | 0.36 |
